# Media coverage as a moderator in the nexus between audit quality and ESG performance: Evidence from China

**DOI:** 10.1371/journal.pone.0312510

**Published:** 2024-10-31

**Authors:** Chun Cai, Saddam A. Hazaea, Maha Faisal Alsayegh, Muskan Sahu, Moodhi Raid, Waleed M. Al‐ahdal

**Affiliations:** 1 School of Accounting, The Center for China’s Governmental Auditing Research, Southwestern University of Finance and Economics, Chengdu, Sichuan, China; 2 Department of Accounting, Faculty of Economics & Administration, King Abdulaziz University, Jeddah, Saudi Arabia; 3 Faculty of commerce, Banaras Hindu University, Varanasi, India; 4 Department of Accounting and Finance, College of Business Administration, Al Yamamah University, Riyadh, Saudi Arabia; 5 Faculty of Business, Economics and Social Development, Universiti Malaysia Terengganu, Kuala Nerus, Terengganu, Malaysia; Zhejiang Sci-Tech University, CHINA

## Abstract

In response to growing pressure on companies to manage and improve their reputation regarding environmental, social, and governance (ESG) issues, the audit is regarded as a vital resource for ensuring ESG risk management, improving transparency, mitigating opportunistic constraints, and guaranteeing accurate reporting. The objective of this paper was to investigate the role of audit quality in improving ESG performance, as well as to examine the role of media coverage represented by ESG controversy score in moderating these relationships. We analyzed 303 Chinese companies with 2,121 observations covering the period from 2017 to 2023. The results suggest that the effects of audit quality as measured by the Big 4 and audit fee on improving ESG performance are positive but not significant. On the other hand, the results reveal that media coverage serves as a positive, albeit non-significant, moderating variable between audit quality measured by the Big 4 and ESG performance, while it has a significant negative effect when audit quality is evaluated based on audit fees. The results indicate that improving ESG performance is significantly linked to auditors intensifying their practices and implementing their work more stringently. More importantly, media coverage is an important additional driver and economic incentive that encourages companies to steer clear of poor ESG-related practices.

## 1. Introduction

Environmental, social, and governance (ESG) reporting has experienced significant growth in the field of corporate reporting over the past two decades. Corporate responsibility has evolved considerably in recent years, shifting from a focus solely on achieving investor goals to balancing the achievement of goals for stakeholders and society as a whole. Recently, there has been a global emergence of investment related to ESG issues, also referred to as "sustainable investment" and "ethical investment" [1]. Therefore, investors aiming to achieve strong corporate governance are embracing the ESG concept linked to ethical investment [2]. Beard [3] and Liu et al. [4] assert that the integration of ESG practices has become a significant and expanding aspect and is one of the fundamental factors contributing to the success of companies. Whyte [5] reported that the implementation of ESG in companies reflects their standing, the effective execution of their operations, their reputation, and their success. The rising demand for companies to focus on ESG issues has resulted in pressure on these firms to invest in social responsibility and disclose the various ESG activities they undertake by companies as they are important matters for stakeholders [6, 7]. In light of these pressures and the Chinese government’s encouragement of companies to prioritize a green economy, transparency, inclusivity, and responsible investment. Chinese companies are striving to enhance their ESG practices. These concepts are deemed essential in modern China [8, 9].

In light of this development, the use and practice of ESG auditing remain a new and emerging topic [10]. Giudice et al. [11] confirm that auditing sustainability reports enhances the reliability and transparency of ESG results, which positively impacts the quality of ESG information and the company’s outputs. Auditing is defined as the process where auditors are expected to identify omissions and errors that may have been made by the company, and then report them [12]. Conversely, ESG auditing is a novel approach aimed at advancing companies’ business and addressing companies’ concerns regarding managing commercial, social, and environmental risks [13]. Therefore, it’s anticipated that auditing can play a crucial role in ensuring ESG reporting. Consequently, the quality of the audit may be a decisive mechanism through which the quality of the company’s strategic choices is improved. It is also one of the fundamental mechanisms for the effective implementation of the initiatives launched by companies on an ongoing basis [14].

Consequently, this study aims to directly investigate the role of audit quality in enhancing ESG performance. This study also It seeks to explore the role of media coverage represented by ESG controversy as a moderator between audit quality and ESG performance. ESG controversy as reported by the media refers to events that can publicly and negatively impact the company’s reputation and expose it to risk. In addition, they undermine investor confidence and can significantly damage the company’s performance [11]. Media coverage is regarded as a crucial tool to improve the disclosure of corporate social responsibility and enhance ESG practices [15]. On the other hand, Burke, Jenna [16] asserts that media coverage possesses a strong capacity to inform and shape public opinion of any organization. However, there is limited research on the role of media coverage in various contexts, particularly its influence on improving the audit and transparency of ESG. Hammami et al. [7] conducted a study on Canadian companies, while Moalla and Dammak [17] studied United States companies. Consequently, there has been little research conducted in emerging countries such as China. Miller, Gregory [18] suggested that the interaction between media coverage and topics related to the economy is an area that warrants further investigation. Furthermore, the findings of Li et al. [19] confirm that the relationship between media coverage and audit is still relatively unexplored. Therefore, there is a pressing need for additional studies to be conducted. This study addresses research gaps in the field of ESG performance by investigating the relatively unexplored area of the impact of audit quality and media coverage in emerging markets. While few studies have focused on Western markets, this research significantly enhances knowledge due to the differences in governance and regulatory frameworks between developed and emerging country contexts. Additionally, it provides empirical evidence that audit fees have a significant positive impact on ESG performance, bridging the gap in understanding the financial aspects of audit quality’s role in ESG. The study also explores the degrees of controversy surrounding ESG, adding a new dimension to the literature by highlighting how media scrutiny affects corporate transparency and accountability in ESG practices.

We selected Chinese firms for this study due to the relatively slow adoption of corporate ESG practices in emerging countries. Therefore, researching a sample of Chinese companies will enhance understanding, enrich the literature, and broaden the application of theories related to ESG issues [20]. In light of the rapid development of the social economy and the increasing intensity of competition, the business environment for companies is becoming more complex, particularly for Chinese firms. As a result, the topic of sustainable development has become one of the most important areas of research [21]. This has prompted us to explore the relationship between auditing and ESG issues using Chinese companies. Additionally, China has unique characteristics that distinguish it from other countries, the most significant being that the data of auditors who sign the company’s reports is publicly accessible [22]. This creates additional pressure for auditors to provide high-quality audits to avoid damaging their reputations. On the other hand, there is careful monitoring of media coverage, ensuring that any event is verified before being reported [23]. Chinese journalists typically avoid taking risks. Consequently, when they report negative news, their credibility is exceptionally high, attracting more attention from readers [24]. The rapid expansion of the social economy has intensified market competition, thereby complicating the business environment for Chinese-listed companies. Therefore, ensuring the sustainable development of Chinese listed companies has become a vital focus of academic research, prompting us to pay closer attention to studying this topic [21].

Using a Refinitiv Eikon database, this study analyzed 303 Chinese non-financial companies with 2121 observations from 2017 to 2023. Following Rajgopal et al. [25] two metrics Big-4 and audit fees, were utilized to measure audit quality. Additionally, we use the aggregated ESG score as the dependent variable. One of the external governance factors represented by media coverage is used as a moderating variable. The results indicate that audit quality as measured by audit fees has a significant and positive impact on ESG performance. Conversely, the results show that media coverage negatively and significantly affects the relationship between audit fees and the enhancement of ESG performance. Furthermore, the results reveal that media coverage has a positive, non-significant effect on the relationship between audit fees and the enhancement of ESG performance.

This paper contributes to the literature by integrating audit quality and media coverage. First, it builds on existing research to provide a more comprehensive understanding of the factors influencing ESG performance. It emphasizes the significance of governance factors such as (internal, audit quality) and (external, media coverage) in promoting sustainable business practices. Second, it provides valuable insights into ESG practices in emerging countries, where regulatory frameworks and corporate governance practices differ markedly from those in developed nations. Third, this study has theoretical and regulatory implications by offering recommendations to decision-makers and regulators on how to enhance ESG performance through improved auditing standards and responsible media practices. In addition, it provides actionable insights for companies striving to meet stakeholder expectations, manage reputational risks, achieve regulatory compliance, and enhance transparency and accountability in corporate reporting. Fourth, by examining the moderating effect of media coverage, this study offers a novel perspective on the dynamics between audit and ESG performance. This adds to the discourse on how public and institutional pressures shape corporate behaviour in the field of sustainability. Additionally, this study explores the role of media coverage as a primary driver and economic incentive that contributes to companies avoiding poor practices related to ESG. Finally, this study is among the few that utilize two measures of audit quality and their impact on enhancing ESG practices to identify the difference in impact. Therefore, the results highlight the importance of audit quality and media coverage for investors to consider when evaluating ESG performance and making investment decisions. It should be noted that while previous research has explored the impact of audit quality on financial performance and governance, few have focused on its effect on ESG performance. The study further adds innovation by integrating two audit quality measures (Big-4 auditors and audit fees), providing a nuanced understanding of their influence on ESG results. Additionally, the paper examines the relatively underexplored domain of media scrutiny, particularly ESG controversy, as a mechanism that influences corporate transparency and ESG practices.

The rest of the paper is organized as follows. Part 2 presents the “Theoretical Background”. Part 3 presents the “Literature Review”. Part 4 presents the variables of the study and the “Methodology”. Part 5 presents the “Results” of the study and the “Discussion”. The last part presents the “Conclusion and Implications”.

## 2. Theoretical backgrounds

In the context of achieving carbon reduction goals and promoting the emerging concept of sustainable development, stakeholders and companies have significantly increased their interest in enhancing ESG performance. Many theoretical perspectives have utilized enriching studies of ESG practices. Barnea and Rubin [26] used agency theory, while, Azizul and Deegan [27] used stakeholder theory. Berrone and Gomez-Mejia [28] used the theory of institutions, Uyar et al. [29] used the theory of legitimacy and Nana et al. [30] used the theory of strong structuring. In this regard, agency theory, legitimacy theory, and institutional theory confirm that companies have strong and close relationships with stakeholders and each group influences and shapes the companies’ disclosure of their responsibility related to ESG [17].

Agency theory provides insights into how corporate managers influence agents’ behaviour through procedures, controls, and incentives consistent with achieving the goals of business interests [31]. It focuses on efforts to minimize information asymmetry and foster consensus among all stakeholders, including management and shareholders [32]. Transparency in ESG plays a role in reducing information asymmetry contributing to enhancing companies’ performance. Thus, companies engaging in and disclosing ESG activities benefit from reduced information asymmetry and reduced agency conflicts [7]. Additionally, agency theory is one of the most important theories in explaining audit quality. It suggests that auditing is a fundamental governance tool that helps ensure a company’s operations are conducted and implemented correctly, thereby enhancing confidence among stakeholders and contributing to achieving the company’s goals and improving performance [33, 34].

The theory of legitimacy posits that fulfilling society’s expectations of a company’s activities is crucial for its survival and competitiveness. Engaging in ESG activities demonstrates the company’s commitment and contribution to meeting stakeholder and societal requirements [7]. Consequently, companies strive to enhance their ESG initiatives to establish their legitimacy. Stakeholder theory elucidates the motivations behind companies’ ESG practices. It emphasizes the need for companies to respond to stakeholder requirements, with disclosure of ESG activities being a critical element in this process [35]. In light of this, companies’ implementation of ESG practices may sometimes lead to conflicts with the interests of certain shareholders [36]. Therefore, audit quality emerges as a mechanism to monitor the execution of ESG activities, reducing information asymmetry, and enhancing the reputation of the companies. This safeguards shareholders’ rights and ensures the proper implementation of ESG initiatives.

**Fig 1:**
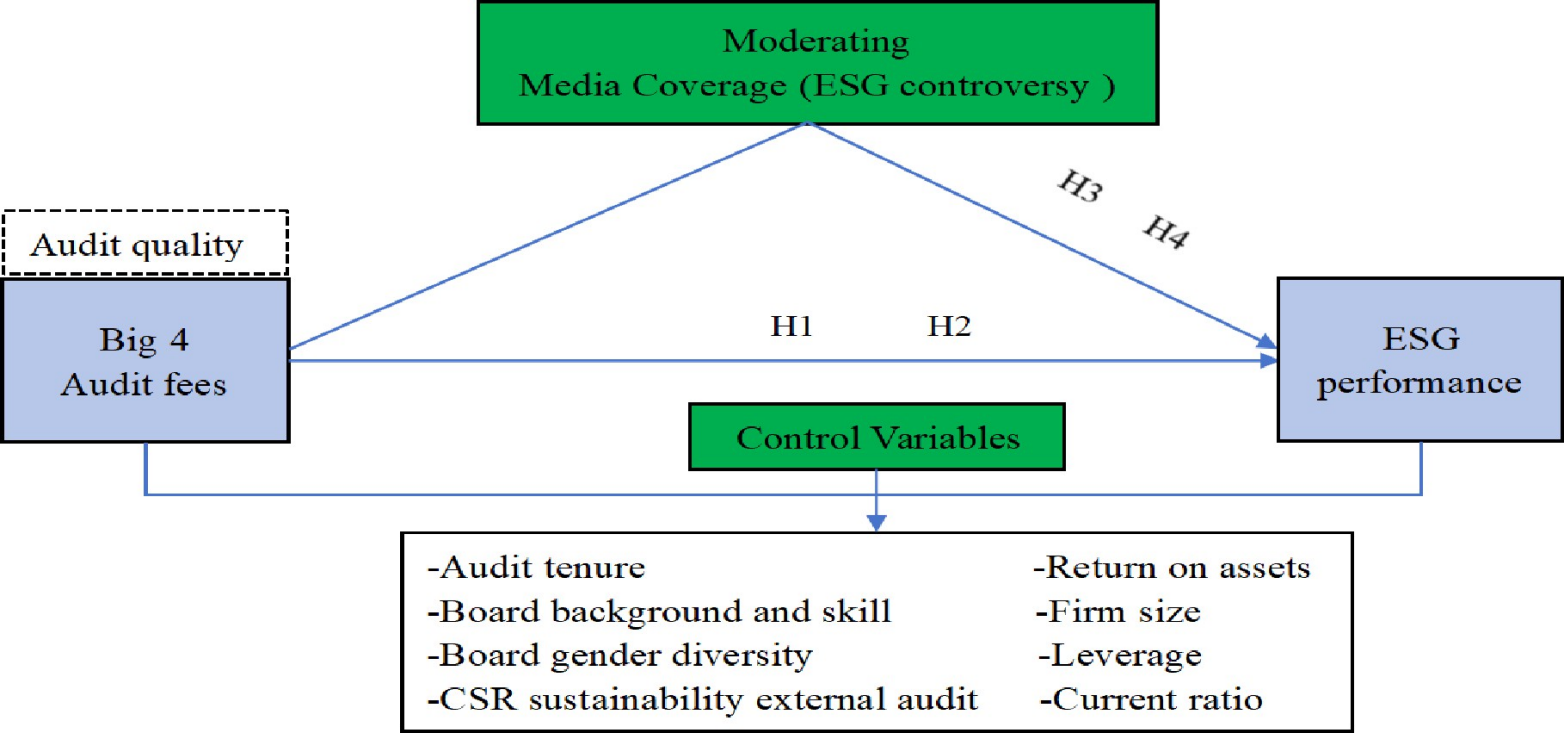
Conceptual framework.

The media agenda-setting theory indicates that media coverage significantly influences companies’ performance regarding ESG activities [37]. It reflects the demand for enhanced ESG performance. Exposure to media coverage serves as a crucial means of enhancing the legitimacy of companies [38]. Finally, media coverage is also regarded as an external governance factor that fosters transparency in the proper execution of ESG activities, protecting shareholders’ rights and enhancing companies’ performance [7, 39]. The framework of the study is shown in Fig 1.

## 3. Literature review

### 3.1. Audit quality and ESG

Agency theory is one of the most significant theories used in auditing [40]. According to the theory developed by Jensen and Meckling [38], auditing is one of the most vital means of reducing information asymmetry, minimizing opportunistic behaviour of any party, including managers and owners, and serves as a crucial means for enhancing ESG practices [11, 41]. According to DeAngelo [42] the audit can be deemed of high quality if it contributes to reducing the risk of misinterpretation and misconduct. While Qader et al. [43] and Taha, Alaa [44] confirm that the quality of auditing plays an important and vital role in ensuring compliance with the regulations, laws and accounting standards governing it, thereby reducing reputational risks and the likelihood of penalties. Thus, audit quality expertise reflects the ability of auditors to discover errors that seriously affect financial reports. Watkins et al. [45] indicate that the quality of auditing aims to enhance confidence, restrict self-exploitation, and provide highly accurate financial reports. Auditing theory indicates that audit quality is based on the effectiveness of the external auditor [46]. Hammami and Zadeh [47] demonstrated that auditing quality effectively helps reduce information asymmetry between company management and stakeholders. Similarly, disclosing non-financial information such as ESG enhances the credibility and reliability of the information obtained by stakeholders [48]. In this context, audit quality is the key factor that enhances the credibility and reliability of the data that company managers share with all stakeholders regarding the company’s status and the level of commitment to implementing ESG activities [47]. In this regard, numerous studies have proposed different measures to assess audit quality. Based on the results of DeFond andZhang [49] the audit quality measures can be categorized into three groups: measures based on outputs, measures based on inputs, and other measures. Therefore, we use two basic metrics to evaluate audit quality in this study: the Big 4 and audit fees. Rajgopal et al. [25] highlight that the most significant input measure of audit quality is audit fees.

### 3.2. Audit quality (Big 4) and ESG

Agency theory is one of the most significant theories applied in the field of auditing [40]. This theory posits that there is a conflict of interest between shareholders and company managers. In this regard, Big 4 auditors are regarded as delivering high-quality auditing processes and practices as a result of the availability of technology, modern auditing methods, and continuous training, and they are not directly linked to clients [50, 51]. Likewise, Bacha et al. [52] and Le and Moor [53] indicate that Big 4 auditors provide high-quality auditing due to their great independence, stemming from their exposure to greater risks if they fail to perform their work accurately. They rely on revenues from the organization rather than directly from a client, exposing them to high risks of litigation and decreasing the cost of debt. The findings of Gong et al. [54] confirm that one of the reasons for the low quality of auditing is the implementation by auditors who are not part of the Big 4. Likewise, the results of Zahid et al. [14] indicate that the Big 4 ensures transparency, reliability, and value in companies’ financial statements, as a result of their commitment to high auditing standards. Ado et al. [55] indicate that the Big 4 seeks to protect its reputation and is diligently working to implement auditing standards without breaching them. On the other hand, Agyei-Mensah [41] highlights that the Big 4 performs significantly in social responsibility. In this regard, Zahid et al. [14] argues that the Big 4 substantial investment in technology and human capital enhances corporate social responsibility more credibly. On the other hand, Xiao et al. [56] argues that the Big 4 can effectively contribute to the preparation of social responsibility reports in a way that strengthens their accountability regarding information and assurance. The results of Saeed et al. [57] confirm that the Big 4 ensures the reliability and credibility of disclosing non-financial information or sustainability reports. In contrast, the findings of Chung et al. [58] indicate that the reporting of ESG practices increases if sustainability reports are audited by the Big 4. Diab Eissa [59] highlights that companies that engage in ESG practices typically hire auditors from the Big 4. This aligns with the results of Zhang and Guo [60] study, which showed that the Big4 have extensive experience in providing ESG auditing services due to their robust internal control systems, highly qualified auditors and clear regulations. These insights are consistent with the assumption of stakeholder theory, which assumes that companies have obligations to all parties, including shareholders, customers, suppliers, and society. Therefore, this theory supports the notion that companies whose ESG reports are audited by the Big 4 tend to demonstrate high ESG performance. As a result, we anticipate that audit quality as measured by the Big 4 will play a crucial role in diminishing information asymmetry between management and stakeholders. Consequently, company managers will disclose both basic and private information about the company to the owners, including disclosure of ESG practices. However, managers require a mechanism and means to validate the credibility and reliability of ESG practices, which may indicate the presence of the Big 4 in these companies. Therefore, we expect that the presence of high-quality audits through the Big 4 will enhance ESG practices by improving the transparency and credibility of information thereby legitimizing it through its contribution to environmental and social initiatives. According to the above, we formulate the following hypothesis.

**H1**: Audit quality, as measured by (Big 4), positively impacts ESG performance.

### 3.3. Audit quality (Audit fees) and ESG

In recent years, significant attention has been directed towards the relationship between audit fees and ESG practices. While audit fees are usually charged for traditional financial reporting audits, some studies suggest a link between audit fees and corporate performance concerning ESG. The findings of Song et al. [61] indicate that better ESG performance results in lower audit fees for Chinese companies. Similarly, the findings of Valls Martínez et al. [62] reveal a significant and widespread association between large audit fees and companies’ implementation of ESG practices. Audits entail a thorough evaluation of the company, including risk assessment, ESG practices, resource allocation, and profitability audit [63]. Therefore, auditors are required to assess ESG reports, in addition to evaluating other company behaviors. This ensures that companies receive support in enhancing their implementation of ESG initiatives and working towards reducing reputational risks [64]. Hence, it can be asserted that higher audit fees, as a measure of audit quality, diminish information asymmetry and improve the transparency of reports. Consequently, the effectiveness of ESG practices and the transparency of companies’ operations are closely linked to higher audit quality, which correlates with increased audit fees ultimately bolstering the legitimacy and reputation of companies [7, 62].

DeFond and Zhang [49] reported that audit fees are among the most significant indicators of audit quality based on inputs. Asante-appiah and Lambert [65] also state that audit fees are one of the key variables in ensuring high quality. The results also demonstrate a strong correlation between fluctuations in audit quality and audit fees. Therefore, companies invest in high audit quality by paying higher fees to enhance the credibility and transparency of their information and reports, including ESG reports [17, 66]. In this regard, Chen et al. [66] argue that paying higher audit fees improves audit quality, which reflects positively on the honesty and reliability of the ESG report. This aligns with the resource-based theory perspective that companies’ disclosure of ESG-related reports is evidence of intangible resources, which requires more audits to investigate and thus higher audit fees [67]. On the contrary, Chen et al. [66] indicate that companies’ involvement in social and environmental activities and initiatives correlates with lower fees. Similarly, Song et al. [61] using a sample of Chinese companies, show a relationship between lower audit fees and better ESG practices. This supports the institutional theory perspective that companies aim to legitimize themselves and meet the goals and expectations of stakeholders by participating in ESG initiatives, so these companies may have lower audit fees as a result of their commitment, participation, and transparency towards stakeholders [67, 68].

Despite the varying results in the literature, we contend that the rise in audit fees stems from increased auditing of the company’s operations, which leads to obtaining results with high transparency and credibility. Therefore, it is anticipated that enhanced audit quality will improve the transparency and credibility of ESG reports. This paper posits that higher audit fees, as an indicator of audit quality, bolster the credibility and transparency of ESG reports. This contributes to gaining investors’ confidence and enhancing the legitimacy of the company’s work. Conversely, in the absence of transparent and highly credible reports, the company’s work may lack legitimacy. We formulate the second hypothesis as follows:

**H2**: Audit quality, as measured by audit fees, positively impacts ESG performance.

### 3.4. Media coverage

Media controversy surrounding a company’s activities and practices significantly impacts the risk assessment and audit efforts of financial reporting auditors. Consequently, auditors perceive an increased risk of errors or fraud when a company is involved in media controversy. This compels them to enhance their audit procedures, implement a more rigorous audit, invest additional time and effort, and consequently charge higher fees. The objective of this response is to ensure the accuracy and reliability of financial statements, financial reports and ESG reports. In support of this, Koh and Tong [69] found that companies exposed to media controversy tend to pay higher audit fees compared to those not involved in or associated with controversial activities. Similarly, the findings of Burke and Hoitash [70] indicate that negative media coverage of a company’s ESG practices is primarily linked to an increase in audit fees, which is a direct result of the heightened risks auditors must address. Additionally, Moalla and Dammak [17] demonstrate that high audit fees promote greater disclosure of companies’ practices regarding ESG activities. Thus, auditors respond to media controversy by intensifying their procedures and ensuring high audit quality to maintain the credibility and reliability of financial statements, thereby leading to increased audit fees.

This study proposes that controversies surrounding ESG can influence the correlation between audit quality and ESG performance. When negative ESG information concerning a company, such as media reports of questionable actions or product scandals, emerges, it can impact ESG practices. This study suggests that ESG controversies play a moderating role in the relationship between audit quality and the disclosure of more transparent ESG information.

According to, Moalla and Dammak [17] the performance and legitimacy of companies are continually assessed based on knowledge, norms, values, and the extent of their contribution to environmental and social activities. All stakeholders, such as investors, customers, and society, regard media coverage as a more credible source of data compared to corporate or analyst reports [71]. The results of the literature indicate that media coverage affects companies’ behaviours and practices, acting as a control tool [72], enhancing transparency and protecting the interests of external agents [73], brooding the impact of regulatory procedures within the company [19], and reducing agency costs [74]. This aligns with media agenda-setting theory, which suggests that media coverage plays a significant role in improving companies’ performance concerning ESG activities. However, media coverage can yield both positive and negative effects [17]. Companies enhance their reputation and legitimacy through ESG practices [75, 76]. Positive media coverage motivates them to improve their ESG-related practices to sustain their legitimacy [17, 77]. Conversely, negative coverage can harm a company’s reputation and lead to a decline in performance, prompting defensive and adaptive mechanisms to restore legitimacy [7, 74]. Following legitimacy theory, exposure to media coverage aids companies in enhancing their ESG-related practices, thereby enhancing reputation and legitimacy [17, 78]. This fosters transparency and delivers information to stakeholders such as investors, consumers, governments, and society [79]. Therefore, increased media pressure is anticipated to further stimulate demand for ESG practices [17], and media coverage is positively correlated with increased ESG practices [80]. Media coverage can function as a mechanism for monitoring auditors’ work, ensuring performance enhancement and improvement behaviour [19]. Media pressure is thought to positively influence auditors’ commitment to independent and efficient work out of fear of damaging their reputation. According to Sharma and Song [81], auditors leverage media coverage to understand how ESG practices affect financial reporting. Moalla and Dammak [17] and Burke and Hoitash [70] indicate a connection between rising audit fees, audit time, greater effort in auditing ESG reports, and reputational risks resulting from negative media coverage. In contrast, Emma et al. [82] confirm that the use of sustainability assurance and auditing is a crucial factor in bolstering the company’s legitimacy and confronting negative media coverage regarding ESG reports. Therefore, media coverage as an external governance factor is expected to play an important role in strengthening the relationship between audit quality and ESG practices. Based on the above, we present the following hypotheses:

**H3**: Media coverage plays a moderating role in the relationship between audit quality (Big4) and ESG performance.
**H4**: Media coverage plays a moderating role in the relationship between audit quality (audit fees) and ESG performance.

### 3.5. Chinese context of audit quality and ESG

With the Chinese government’s emphasis on green finance, ESG and sustainability reports have seen significant development. According to Zahid et al. [83] the continued growth of ESG activities in China is attributed to two main factors: first, the commitment of Chinese organizations and the government to promoting green finance to ensure harmonious economic and environmental development in the world’s second-largest economy. Second, China is working to attract global investment, so it is mandating listed companies to disclose ESG activities [84]. Despite the progress in the application and implementation of ESG activities by Chinese companies, specific standards for evaluating these practices are still lacking alongside the necessity for companies to conduct independent audits of ESG reports to ensure the enhancement of information. In addition, accounting firms should develop and enhance auditor talent to ensure effective auditing of ESG reports. This can be achieved by appointing highly qualified auditors who can practice effective auditing and identify risks through ESG reports [60]. In this regard, the results of Wang et al. [85] confirm that promoting robust development in the Chinese economy is linked to enhancing ESG performance and ensuring this performance through auditing. Effective scrutiny of ESG reporting adds credibility, enhances investor confidence, and supports sustainable economic growth. Finally, the results of Shen et al. [9] confirm that the rise in regulations and regulatory procedures in China, which call for the engagement of Chinese companies in ESG activities was significantly associated with increased audit implementation. This regulatory impetus emphasizes the critical role of audit quality in enhancing the credibility and reliability of ESG reporting.

## 4. Research methodology

### 4.1. Sample

We utilized the Refinitiv Eikon database to gather the sample used in this study, which comprises 377 companies with a total of 2,639 observations during the period from 2017 to 2023. Following Zahid et al. [83] we excluded all companies operating in the insurance and banking sectors due to the unique regulatory environments, business models, and financial characteristics of these entities, which can significantly differ from those of non-financial companies. Additionally, companies with missing data were removed. The final dataset includes 303 firms, corresponding to 2121 observations. Table 1 and S1 Appendix provide information on the distribution of the sample of companies used in this study based on the annual distribution and industry type.

**Table 1:** Sample selection.

Sample selection	Firms	Observation	%
Initial sample after removing the firms with missing data	377	2639	100
Financial firms (Banks and insurance)	74	518	19.63%
Final sample	303	2121	80.37%

**Source (s)**: Authors’ design

### 4.2. Variable measurements

#### 4.2.1. Independent variables

Following Rajgopal et al. [25] Hay [86] and Krishnan et al. [87] two variables were utilized to measure audit quality (Big 4 and audit fees). The Big 4 firms (Deloitte, KPMG, Ernst & Young, and PWC) are typically engaged to ensure the quality of information through external audit, thereby enhancing effective quality assurance [83]. These companies offer audit services of the highest quality and accuracy to maintain their reputation and established brand [88]. Using this scale, audit quality is defined as a binary variable that takes the value of “1” when a Big 4 firm is employed, and “0” otherwise. In addition to using the Big 4, we also consider audit fees (AUDFEE) as a measure to evaluate audit quality. This measure assesses how well audit fees correspond with the services provided to high-risk clients [86, 87]. We use audit fees as a measure of audit quality because it enables us to capture subtle differences in quality for all companies as a continuous variable. Additionally, high fees are an indicator of providing highly reliable services that help mitigate the risks associated with financial misconduct such as fraud and working to document high-quality financial reports [49]. Therefore, AUDFEE which is the amount the client company pays to the audit firm can be expressed using the natural logarithm.

#### 4.2.2. Dependent variable

Following Moalla and Dammak [17] ESG transparency is used as a dependent variable because it is multidimensional, and derived from a variety of financial and non-financial indicators. ESG practice assessments serve as a mechanism through which sustainability reports from various companies can be collected, and the results of their research can be combined for the study [83]. The ESG score in the Refinitiv Eikon database is calculated on a scale from 0 to 100. The score ranges from a minimum of 0 to a maximum of 100. Al-ahdal et al. [89] reported that Refinitiv Eikon provides objective and systematic ESG information, which helps investors integrate various social responsibility characteristics when making investment decisions. According to, Refinitiv Environmental, S and G [90] the ESG score is divided into 12 levels, starting from “A+” as the highest score, which ranges from 91 to 100, and down to the lowest level “D-,” which spans from 0.0 to 0.08. While the 12 scores are divided into 4 levels, where the score is less than 0.25 in group (D), it has a relative performance index for ESG standards related to transparency, and the ESG index is weak. While the second group represents group (C), the score is greater than 0.25 and less than 0.50. Here, the transparency and ESG index are satisfactory. B group represents the third level in which the ESG score is greater than 0.50 and less than 0.75. This indicates that the transparency and performance of companies regarding ESG are good. Finally, the fourth level, represented by group (A), includes scores greater than 0.75 to 1, indicating high and excellent transparency and company performance. Following Hammami and Hendijani Zadeh [7], Moalla and Dammak [17], and Refinitiv Environmental, S and G [90] we utilized the combined score of the ESG elements to ensure a comprehensive and integrated evaluation of the company. This evaluation is based on all the information included in the corporate pillars.

#### 4.2.3. Moderating and controlling variables

We utilize media coverage as a moderating variable in this study, which is measured through the ESG controversy score. The controversy surrounding ESG issues includes negative news related to company product scandals and undesirable activities in the media that adversely affect the market value [91, 92]. According to, Refinitiv Eikon Datastream [93] the score is calculated by considering negative media coverage such as legislative disputes, fines, and lawsuits filed against companies. The final result is derived from assessing 23 diverse controversial topics about ESG activities and practices, and this is measured by a percentage, which reflects the level of disagreement or controversy that occurred in the company during a specific period, usually a financial year [94]. Finally, following Hammami and Hendijani Zadeh [7], Moalla and Dammak [17] and Zahid et al. [83] we have included some factors as controlling variables that have an impact on the relationship between audit quality and ESG performance. Table 2 presents the definition of all variables used in this paper.

**Table 2:** Definition of variables.

Variable	Symbol	Definition	Source
***Independent variable***			
Big 4	BIG4	A dummy variable is assigned a value of 1 when a Big 4 firm is used, and 0 when it’s not.	[95]
Audit fee	AUDFEE	The audit fees that the client company pays to the audit firm can be represented by natural logarithm	[96]
***Dependent variables***			
ESG		Indicate the natural logarithm of the transparency of the ESG score,	[7]
***Moderator variable***			
Media coverage	MEDCO	The ESG controversy refers to the presence of negative news about the company’s ESG practices. It also includes the media attention that the company receives as a result of this controversy.	[17]
***Control variables***			
Audit tenure	AUDTE	A measure of the duration that the auditor spends conducting a business audit for the company.	[50]
Board special background and skill	BBS	Percentage of the extent to which company board members have specific backgrounds and experience in the industry	[97]
Board gender diversity	BGD	A metric that indicates the proportion of women and men serving on the board of directors of the company.	[98, 99]
CSR sustainability external audit	CSRAUD	Number of companies that have an external auditor to review a CSR sustainability report	[100]
Return on assets	ROA	Net income divided by total assets	[7]
Firm size	FSIZE	A measure of the logarithm of total assets	[89, 98]
Leverage	LEV	Total long-term debt divided by the total equity	[101]
Current ratio	CUR	A metric to gauge financial downturn by calculating the ratio of current assets to current liabilities.	[102]

### 4.3. Empirical model

Based on the theoretical foundation of this study and the description of the variables, we established the study model using four different models. Model **1** demonstrates the direct relationship between audit quality (BIG4) and ESG performance. Model **2** outlines the direct relationship between audit quality (AUDFEE) and the transparency of ESG practices. Through this analysis, we seek to determine how the influence of audit quality on ESG transparency varies or remains consistent based on the use of two different variables.

**Model 1 and Model 2**: Direct effect (BIG 4, AUDFEE and ESG)

***ESG****it = β0+ β1 BIG4 it + β2 AUDFEEit +β3 AUDTE it + β4 BBSit + β5 BGDit + β6 CSRAUD it + β7 ROA it + β8 FSIZE it + β9 LEV it + β10 CURit + YEAR_dummiesit + INDUSTRY_ dummiesit + COMPANY_dummiesit + ε it* **(1)**

In addition to that we used media coverage, measured by ESG controversy as a moderating variable (BIG4 * MedCo *it* and AUDFEE* MedCo *it*) and it was used in models 3 and 4.

**Model 3 and Model 4**: The moderating effect of **MedCo** (BIG4 and AUDFEE)

***ESGi***it = β0+ β1 BIG4it + β2 BIG4 * MedCoit + β3 AUDFEE * MedCoit + β4 AUDTEit + β5 BBSit + β6 BGDit + β7 CSRAUDit + β8 ROAit + β9 FSIZEit + β10 LEVit + β11 CURit + YEAR_dummiesit + INDUSTRY_ dummiesit + COMPANY_dummiesit + εit **(2)**

We employ the stepwise estimation method to develop the model and include variables to mitigate any potential bias caused by omitted variables. We have also constructed earlier models (1–4) that encompass all variables. It is important to note that the symbol i denotes the firm, t signifies the years, and ε represents the error term. Finally, to prevent common endogeneity problems resulting from the passage of time across industries and companies, we include year, industry, and company dummies.

### 4.4. Ethics approval

This article does not contain any studies with human participants or animals performed by any of the authors.

## 5. Results and discussion

### 5.1. Descriptive statistics

Using STATA version 17 software all statistical analyses, tests, and regressions were conducted. The descriptive statistics presented in Table 3 indicate a moderate level of ESG practices 39.58, among the sampled companies. This score suggests that the ESG practices of Chinese companies are acceptable and satisfactory [90]. This score differs from the results reported by Zhang et al. [96] who discussed the relationship between ESG practices and audit fees using a sample of Chinese companies from 2011 to 2020. They reported a score of 20.93. Therefore, the ESG score shown in Table 3 indicates that Chinese companies require further improvement in their ESG practices to ensure and improve their informational capability. The results also indicate a significant discrepancy between the ESG practices implemented by Chinese companies. The lowest value for the ESG score reached 0.99, while the highest value reached 93.31. These findings align with Zahid et al. [83] and Zhang et al. [84] which confirm that the degree of ESG practices in Chinese companies is lower than in the United States and Europe. This indicates a lack of focus and incentives to encourage companies to disclose ESG practices. In addition, the variable exhibits a standard deviation of 17.36, which is less than the mean. This indicates the diversity of the sample as well as the different levels of ESG practices across the companies in the sample.

**Table 3:** Descriptive statistics.

Variable	Obs	Mean	Std. Dev.	Min	Max
**BIG4**	2121	0.17	0.38	0	1
**AUDFEE**	2121	12.96	1.19	8.55	16.96
**ESG**	2121	39.58	17.36	0.99	93.31
**MEDCO**	2121	97.57	11.94	1.44	100
**AUDTE**	2121	5.67	3.97	1	24
**BBS**	2121	36.74	22.79	0	55.14
**BGD**	2121	51.29	28.15	2.18	99.95
**CSRAUD**	2121	0.06	0.24	0	1
**ROA**	2121	0.05	0.26	-9.92	1.55
**FSIZE**	2121	9.60	0.75	6.68	12.20
**LEV**	2121	0.20	0.22	0	3.53
**CUR**	2121	4.94	67.48	.035	2765.90

Table 3 also indicates that a few Chinese companies (mean = 0.17) are audited by the Big 4. These findings align with Zahid et al. [83] which shows a lack of commitment from Chinese companies toward Big 4 audit services. This suggests that Chinese companies have relatively low-level auditing operations despite the perception that Big 4 audits are of high quality due to the experience, independence, and professionalism of their members as well as the credibility and reliability of their reports [53, 57]. The average score of audit fees for these firms stands at 12.96, characterized by a low standard deviation of 1.19, indicating that most of these companies undergo costly audit processes [17]. We standardize the audit fees by converting them to thousands of US Dollars and calculating the natural logarithm of the fees paid by client companies to audit firms or auditors. It is important to note that, the level of financial statement authentication, reliability of internal control, and the assessment of fraud risk including fraudulent transactions are some of the elements that determine the range of AUDFEE, values from the minimum to the maximum. Furthermore, the sample firms’ media coverage of ESG practices, or MECOV, has a mean value of 97.57 and a standard deviation of 11.94. The average percentage of women serving on the board is 51.29%, while the proportion of board members with significant expertise in finance or specialized industry knowledge is 36.74%. Additionally, the average audit tenure AUDTE, representing the duration of the auditor’s engagement with the company, stands at 5.67. Moreover, the average proportion of companies with an external auditor for their CSR sustainability report is 0.06. This indicates that there are a limited number of Chinese companies that have external auditors to review CSR reports. The average ROA is 0.05, and the average firm size is 9.60. Furthermore, the mean of LEV is 0.20 and the mean of CUR is 4.94.

### 5.2. Correlation

S2 Appendix presents the findings of a correlation analysis. The results indicate a positive (0.278***) and significant correlation (p-value <0.01) between audit fees and ESG. This suggests that companies paying higher audit fees are more likely to provide thorough and transparent disclosures regarding ESG activities [96]. Moreover, the correlation analysis reveals a significant negative correlation between MEDCOV and ESG (-0.104***). This indicates that companies facing more controversies related to ESG matters tend to exhibit lower levels of ESG practices. In contrast, firms with higher ESG practices are less vulnerable to controversies highlighted in media coverage. Regarding the control variables, AUDTE shows a positive and significant correlation with one audit quality measure by Big 4 firms. This suggests that extended AUDTE may enhance audit quality, as evidenced by the increased preference for Big 4 auditors. This contradicts the findings of previous studies, which show that AUDTE is negatively related to audit quality [50, 103]. Additionally, the relationship between AUDTE and MEDCO is negative, indicating that companies experiencing more ESG-related controversies tend to have shorter AUDTE. Furthermore, FSIZE demonstrates a positive association with ESG practices, suggesting that larger firms are more likely to be transparent and disclose information regarding ESG issues. Moreover, the presence of external CSR audits shows a positive association with ESG practices, suggesting that companies tend to disclose information on ESG issues when they undergo external audits for CSR reporting.

The results indicate that all correlation values are below the multicollinearity threshold of 0.80, suggesting no concerns about multicollinearity. The highest value reached 0.66 for the control variable FSIZE and its relationship with AUDFEE [104]. Conversely, we utilized the VIF, and the results indicate that all values are below the critical level of 10 [105] and also below 5 [106]. We also use Tolerance (TOL) known as the inverse of VIF, to evaluate the linear relationship. TOL values below 0.1, approaching 0, indicate the possibility of a linear relationship between the study variables. In contrast, TOL values close to 1 suggest no linear relationship between the study variables. The results presented in S2 Appendix indicate that all TOL values are close to 1, providing further evidence that there is no multicollinearity issue among all variables of our study [107–109].

After conducting individual tests on effects, residual normality, heteroscedasticity, and autocorrelation, we have identified issues in several areas. Based on the results, we found non-normality of residuals as indicated by skewness and kurtosis tests, heteroscedasticity based on the Breusch–Pagan/Cook–Weisberg tests, and autocorrelation detected through the Wooldridge test across all models. Therefore, to address issues related to normality, heteroscedasticity, and autocorrelation of errors in estimating the presented models, the generalized least squares (GLS) method is more suitable [17].

### 5.3. Generalized least squares (GLS)

#### 5.3.1.Audit quality and ESG

Using the Big 4 and audit fees to measure the direct effect of audit quality on the ESG performance in Chinese companies, we analyzed two models (Model 1) and (Model 2) (see Table 4). The results indicated a positive effect of the Big 4 on ESG practices but it was not statistically significant at its value (Coeff = 0.391). This suggests that the Big 4 does not significantly impact enhancing ESG practices in Chinese companies. These results confirm the findings from the descriptive test in Table 3, which indicated that few Chinese companies use Big 4 services. Instead, they rely on other auditing firms. These results are consistent with the findings by Zahid et al. [83] who also found no effect of audit quality as measured by the Big 4 on enhancing ESG. This result contradicts the view of Dakhli [104] who emphasizes the importance of the Big 4 in enhancing ESG practices. These results align with the institutional theory perspective. Therefore, in the Chinese context, regulatory pressures may not prioritize the use of Big 4 auditors. Instead, firms rely on other audit firms. However, these results contradict the view of legitimacy theory, which suggests that companies seek to legitimize their operations through practices that are acceptable to society [7]. So, the use of Big 4 auditors may be seen as part of that.

**Table 4:** Regression results using generalized least square.

	Model 1	Model 2	Model 3	Model 4
BIG4	0.391		2.421	
	(0.782)		(1.232)	
AUDFEE		0.392		2.496***
		(1.481)		(2.838)
MEDCO			0.012	0.322**
			(1.090)	(2.568)
MEDCO_BIG4			-0.022	
			(-1.100)	
MEDCO_AUDFEE				-0.022**
				(-2.521)
AUDTE	0.033	0.031	0.037	0.028
	(0.583)	(0.541)	(0.636)	(0.475)
BBS	-0.251***	-0.252***	-0.250***	-0.249***
	(-33.777)	(-33.798)	(-33.280)	(-32.777)
BGD	0.008	0.008	0.007	0.007
	(1.068)	(1.045)	(0.979)	(0.973)
CSRAUD	3.156***	3.185***	3.044***	2.887***
	(4.767)	(4.816)	(4.518)	(4.271)
ROA	-1.000***	-0.964***	-0.989***	-0.920***
	(-3.610)	(-3.458)	(-3.506)	(-3.257)
FSIZE	2.277***	1.977***	2.284***	2.050***
	(4.083)	(3.373)	(4.017)	(3.496)
CUR	-0.005**	-0.005**	-0.005**	-0.005**
	(-2.574)	(-2.557)	(-2.569)	(-2.556)
LEV	-0.162	-0.168	-0.162	-0.101
	(-0.256)	(-0.265)	(-0.254)	(-0.157)
Year effect	Included	Included	Included	Included
Industry effect	Included	Included	Included	Included
Company effect	Included	Included	Included	Included
Constant	34.599***	32.202***	33.371***	0.369
	(6.208)	(5.521)	(5.763)	(0.027)
Observations	2121	2121	2121	2121
Wald chi-square	34714.21	34791.01	34556.31	34602.87
Prob>chi-square	0.000	0.000	0.000	0.000

Note

* *p <* .*1*

** *p <* .*05*

*** *p <* .*01*.

Additionally, in Model 2, the results indicate that the effect of audit fees (AUDFEE) on ESG practices is positive but statistically insignificant (Coeff = 0.392). These findings suggest that companies do not recognize the importance of comprehensive and professional auditing practices in showcasing their dedication to implementing ESG principles and fulfilling the expectations of all stakeholders. This outcome does not support the perspective of agency theory, which asserts that enhancing audit quality contributes to restricting opportunistic corporate behaviour, alleviating information asymmetry, and enhancing ESG practice [41, 110, 111]. Thus, H2 is rejected. Logically, the increase in audit fees paid by companies reflects their intention to improve the transparency and credibility of financial reporting which ignores ESG reporting. These results contradict with Chen et al. [66] those who confirm that companies that pay higher audit fees seek to enhance the credibility and transparency of their reports in a way that instils the confidence of investors and stakeholders. Likewise, Zhang et al. [96] assert that companies that disclose their ESG practices pay high audit fees. Therefore, companies that wish to demonstrate a high degree of disclosure of ESG practices face high audit risks, necessitating higher fees. This is consistent with the risk premium theory, which indicates that increasing audit fees contributes to a high allocation of resources and enhances the credibility of auditors’ high professional and ethical commitment. This, in turn, affects the promotion of the disclosure of ESG practices by companies [112].

Following Hammami and Hendijani Zadeh [7], Moalla and Dammak [17] and Zahid et al. [83] we have included several factors as controlling variables that have an impact on the relationship between audit quality and ESG practices. To address endogeneity issues, we employed panel GLS regression with fixed effects (year, industry, and company). The results indicate that the presence of a CSR external auditor (CSRAUD) has a positive and significant impact at a 1% level of significance (p-value < 0.01) across all models. These findings suggest that companies benefit from the expertise, support, credibility, alignment with stakeholder expectations, risk management, and the competitive advantage provided by CSR external auditors, leading to enhanced ESG practices. These auditors possess specialized knowledge and professional skills in evaluating and auditing ESG-related issues, which contributes to the transparency and accuracy of ESG practices. Additionally, when considering all the models, FSIZE shows a positive and statistically significant (p-value < 0.01) influence on ESG performance. These findings suggest that larger firms tend to exhibit greater ESG practices. This aligns with the findings of Chung et al. [58] which indicate that large companies whether voluntarily or mandatorily tend to have greater ESG practices. The results of the remaining control variables are presented in Table 4.

#### 5.3.2. Audit quality, media coverage, and transparency of ESG

We examine the role of media coverage as a moderating variable between audit quality and the enhancement of ESG practices. In our analysis, we performed Models 3 and 4 (refer to Table 4) and introduced interaction variables involving both audit quality measures: MEDCO*BIG4 and MEDCO*AUDFEE.

Model 3 reveals that the interaction term MEDCO*BIG4 has a positive effect (Coef = 0.05) but it is not statistically significant concerning ESG. This suggests that media coverage doesn’t play a significant moderating role between BIG4 and the transparency of ESG. However, Model 4 shows that the interaction term MEDCO*AUDFEE has a negative and significant relationship at the 5% level. This means that media coverage affects the association between audit quality measured by audit fees and ESG. This indicates that, in light of widespread media coverage, the positive effects of audit quality on enhancing ESG practices are reduced. This highlights the necessity and importance of companies preparing accurate and transparent reports that mitigate reputational risks resulting from negative media coverage. Therefore, it is likely that when the media looks widely at a company’s practices, deficiencies will be revealed in ESG reports or problems will occur in high-quality auditing practices. So, this intense focus by the media may reduce the anticipated positive impact of audit quality on the transparency and disclosure of companies about their ESG-related activities. Our third hypothesis is partially supported. While audit fees support our hypothesis, BIG4 does not, as media coverage does not moderate the relationship between BIG4 and ESG practices in our sample of Chinese companies. This result is consistent with [17]. This indicates that increased media coverage enhances companies’ desire to commit to implementing high-quality auditing practices, which positively reflects on their ESG performance. Therefore, it is expected that negative media coverage adversely affects the reputation of companies, encouraging auditors to exert more effort and time to ensure high audit quality, which entails higher fees. These findings are consistent with the legitimacy theory. The theory posits that companies will work to disclose their positive activities and responsibilities related to ESG more transparently to ensure and strengthen their legitimacy and enhance their reputation, which may have been affected by negative news [7]. Moreover, these results are consistent with the media agenda-setting theory view, which emphasizes the significant role of media coverage in increasing the salience of a particular issue thereby enhancing transparency and accountability in companies [37, 113].

### 5.4. Additional analysis

#### 5.4.1.Robustness check

*5*.*4*.*1*.*1*. *Generalized Method of Moments (GMM)*. To address endogeneity concerns, we employed GMM as illustrated in Table 5. The application of GMM is one of the most effective statistical methods used to investigate key sources of endogeneity, such as simultaneity, dynamic homogeneity, and unobserved heterogeneity [114, 115]. The results presented in Table 5 reveal that the p-values of AR (2) are non-significant across all models (M1 to M4). Therefore, these values are not influenced by second-order autocorrelation. Furthermore, the GMM indicates a significant positive moderating effect of media coverage on the relationship between audit fees and ESG practices. This result contrasts with the main analysis shown in Table 4. suggesting that further investigation in future research is warranted. Nevertheless, the overall results shown in Table 5 closely align with those of the main analysis presented in Table 5, indicating the reliability and robustness of our findings. The insignificant p-values of the Hansen statistic test for instruments across all models confirm the validity of the instruments utilized. On the other hand, the GMM approach effectively addresses the issues of simultaneity, endogeneity, and unobserved heterogeneity, which are common challenges when using audit fees as a proxy for audit quality in investigations of the concerns of using Big4 and audit fees as indicators of audit quality. Therefore, the results from GMM presented in Table 5 align closely with the main findings in Table 4, reinforcing the robustness of our model. Thus, using audit fees as a proxy for audit quality is particularly significant in the context of business scale. In this regard, larger companies tend to incur higher audit fees due to the complexity and scope of their operations [116]. To differentiate audit fees from merely reflecting business scale, our models control for firm size (FSIZE) and other relevant variables such as industry and company effects. Additionally, we employed fixed effects models and incorporated (CSRAUD), whose positive and significant results further validate the use of audit quality proxies. Therefore, while audit fees may reflect business scale to some extent, our inclusion of firm size and industry-specific control ensures that audit fees still provide meaningful insight into audit quality. Finally, the consistency of results between the GMM approach and the main analysis further supports the robustness of Big 4 and audit fees as effective proxies for audit quality, reinforcing their role in ESG performance evaluation.

**Table 5:** Regression results using GMM.

	Model 1	Model 2	Model 3	Model 4
L.ESG	0.370***	0.335***	0.338***	0.355***
	(9.235)	(8.598)	(8.783)	(7.643)
BIG4	3.276		6.795	
	(0.763)		(0.589)	
AUDFEE		12.493***		-43.925**
		(2.778)		(-2.442)
MEDCO			-0.071	-6.844**
			(-0.923)	(-2.568)
MEDCO_BIG4			-0.067	
			(-0.614)	
MEDCO_AUDFEE				0.464**
				(2.550)
AUDTE	1.796**	0.425	0.401	0.217
	(2.531)	(0.573)	(0.540)	(1.291)
BBS	-0.160***	-0.466***	-0.468***	-0.239***
	(-5.267)	(-3.555)	(-3.604)	(-6.582)
BGD	0.124***	-0.038	-0.082	0.041**
	(2.714)	(-0.386)	(-1.011)	(2.441)
CSRAUD	13.716***	13.287***	19.783***	8.906***
	(7.012)	(3.260)	(5.930)	(3.670)
ROA	1.584	4.510	-0.410	-1.062
	(1.274)	(1.475)	(-0.314)	(-0.920)
FSIZE	5.916***	-16.936***	-4.062	8.824***
	(5.544)	(-2.971)	(-0.884)	(4.239)
CUR	0.003	-0.004*	-0.002	-0.001
	(1.561)	(-1.766)	(-1.016)	(-0.610)
LEV	10.914*	-44.479*	-48.281**	-6.299
	(1.887)	(-1.810)	(-2.096)	(-1.412)
Year effect	Included	Included	Included	Included
Industry effect	Included	Included	Included	Included
Company effect	Included	Included	Included	Included
Constant	-570.704***	-161.836	-144.057	612.586**
	(-3.208)	(-0.763)	(-0.803)	(2.399)
No. of groups	303	303	303	303
No. of instruments	55	50	52	61
F-stats	15.25	11.03	16.87	933.99
Prob>F	0.000	0.000	0.000	0.000
AR(1) p-value	0.000	0.000	0.000	0.000
AR(2) p-value	0.948	0.316	0.514	0.574
Hansen test	0.164	0.53	0.332	0.168

Note

* *p <* .*1*

** *p <* .*05*

*** *p <* .*01*.

## 6. Conclusion and implications

In light of China’s growing commitment to promoting sustainable development, particularly in recent years, interest in studying ESG issues has surged. In this regard, companies are increasingly striving to incorporate ESG standards in their core activities and investments to ensure they meet investors’ expectations and enhance their reputation in society. Using Refinitiv Eikon to extract data on audit quality, media coverage, and their impact on ESG performance. We investigate the role of audit quality in enhancing ESG performance in the Chinese market, using media coverage as a moderating variable. The results show that the effect of audit quality as measured by the Big 4 on improving ESG performance is positive but not significant. We argue that this is due to the limited engagement of Chinese companies with Big 4 services (mean = 0.171) and their reliance on other audit firms. Similarly, there is an insignificant positive effect when measuring audit quality using audit fees. Conversely, the results show that media coverage plays a positive, non-significant role as a moderating variable between audit quality measured by the Big 4 and ESG transparency, while it has a significant negative effect when measuring audit quality by audit fees, likely due to its association with negative media coverage. The results suggest that enhancing ESG performance is significantly linked to auditors intensifying their practices and implementing their work more stringently. More importantly, media coverage serves as a significant driver and economic incentive that contributes to companies avoiding poor practices related to ESG. Therefore, companies should strengthen their auditing standards and practices to ensure rigorous auditing, which will positively influence ESG performance and provide long-term benefits to companies. Additionally, companies should manage the risks associated with media coverage of ESG activities and develop proactive strategies to address any ESG-related discrepancies and enhance their reputation by implementing ESG initiatives. Although this study provides broad and rich empirical evidence to investigate the role of audit quality and media coverage in enhancing ESG performance, there are important areas for future research, such as investigating the role of internal audit quality. Additionally, the effect of audit quality on improving disclosure can be investigated through various evaluations of ESG systems. We expect the results of this study to enhance the achievement of sustainable development goals and strengthen the ESG framework in China to promote investment and the development of green business practices. Finally, it can be concluded that ESG performance can be enhanced through the implementation of rigorous audits of sustainability reports, in addition, to media pressure, which can positively influence the company’s reputation and investments.

### 6.1. Managerial implications

Based on the results of the study, the theoretical and managerial implications can be presented as follows:

- Due to growing investor interest in companies that engage in ESG activities, Chinese companies must integrate these principles into their operations. Our sample results indicated a limited number of companies using the Big 4, indicating a positive but not significant effect of the Big 4 on enhancing ESG practices. This suggests that Chinese companies should focus on improving ESG outcomes by expanding their use of reputable Big 4 services. This can enhance the company’s reputation and attract more investors.
- Given the overall trend towards achieving sustainability, Chinese organizations and the government should ensure the standardization of ESG standards and encourage the use of governance tools, such as Big 4 services, to enhanced reporting transparency and attract international investors.
- The Chinese government should strive to improve the ESG classification and implementation scores of listed companies by taking steps and enacting incentive legislation that encourages companies to adopt ESG practices. Moreover, the Chinese government can develop an audit and assurance system that reliably and independently identifies and classifies various ESG activities.

### 6.2. Practical implications

The findings of this study also have practical implications, the most important of which are:

- There is a need to improve the disclosure of Chinese companies’ adoption and implementation of ESG activities. This requires regulators to require companies to issue ESG reports transparently and comprehensively.
- To enhance the reliability of companies’ reports regarding ESG activities, these companies’ reports must be audited by independent auditing firms thereby enhancing investor confidence.
- Regulators should standardize the criteria for evaluating ESG reports, and this is fundamentally linked to the global trend towards achieving sustainability. Therefore, clear mechanisms and policies can be developed for companies to follow and implement to ensure that they reliably engage in ESG activities.
- The study results showed a significant impact of audit fees on ESG performance, so companies should prioritize this as a crucial factor to ensure ESG reporting, which means conducting more independent and thorough audits.
- Companies should actively engage and participate in media strategies to enhance their reputation. In return, they should continuously monitor the negative effects of media coverage and address challenges to ensure their reputation is enhanced and their efforts to achieve sustainability are demonstrated.

**Note**: *The results of skewness and kurtosis*, *Breusch–Pagan/Cook–Weisberg*, *and Wooldridge tests are available upon request*

## Supporting information

S1 Appendix Distribution of the sample by industry. (DOCX)

S2 Appendix Pairwise correlations. (DOCX)

S1 Data (XLSX)
