# Supplementary material for: Media coverage as a moderator in the nexus between audit quality and ESG performance: Evidence from China
Source: PLoS One. 2024 Oct 31;19(10):e0312510. doi: 10.1371/journal.pone.0312510 (PMC11527248; doi:10.1371/journal.pone.0312510)
Supplement: S1 Appendix — (DOCX) [file pone.0312510.s001.docx]

Appendix 1

Distribution of the sample by industry.

| **Sector** | **Freq**. | **%** |
| --- | --- | --- |
| Advanced Medical Equipment & Technology | 14 | 0.66 |
| Biotechnology & Medical Research  Business, Social Services | 35  35 | 1.65  1.65 |
| Communications & Networking | 91 | 4.29 |
| Computer Hardware | 56 | 2.64 |
| Electric Utilities | 56 | 2.64 |
| Electronic Equipment & Parts | 175 | 8.25 |
| Financial Technology (Fintech) | 21 | 0.99 |
| Healthcare Facilities & Services | 84 | 3.96 |
| Household Electronics | 14 | 0.66 |
| IT Services & Consulting | 98 | 4.62 |
| Independent Power Producers | 56 | 2.64 |
| Integrated Hardware & Software | 14 | 0.66 |
| Integrated Telecommunications Services | 35 | 1.65 |
| Medical Equipment, Supplies & Distribution | 63 | 2.97 |
| Miscellaneous Educational Service Providers | 21 | 0.99 |
| Multiline Utilities | 7 | 0.33 |
| Natural Gas Utilities | 14 | 0.66 |
| Online Services | 168 | 7.92 |
| Pharmaceuticals | 448 | 21.12 |
| Phones & Handheld Devices | 21 | 0.99 |
| Real Estate Services | 315 | 14.85 |
| Semiconductor Equipment & Testing | 35 | 1.65 |
| Semiconductors | 105 | 4.95 |
| Software | 105 | 4.95 |
| Water & Related Utilities | 28 | 1.32 |
| Wireless Telecommunications Services | 7 | 0.33 |
| **Total/Average** | **2121** | **100.00** |
