# Supplementary material for: Media coverage as a moderator in the nexus between audit quality and ESG performance: Evidence from China
Source: PLoS One. 2024 Oct 31;19(10):e0312510. doi: 10.1371/journal.pone.0312510 (PMC11527248; doi:10.1371/journal.pone.0312510)
Supplement: S2 Appendix — (DOCX) [file pone.0312510.s002.docx]

Appendix 2

Pairwise correlations

| **Variables** | **(1)** | **(2)** | **(3)** | **(4)** | **(5)** | **(6)** | **(7)** | **(8)** | **(9)** | **(10)** | **(11)** | **(12)** |
| --- | --- | --- | --- | --- | --- | --- | --- | --- | --- | --- | --- | --- |
| **(1) BIG4** | 1.000 |  |  |  |  |  |  |  |  |  |  |  |
|  |  |  |  |  |  |  |  |  |  |  |  |  |
| **(2) AUDFEE** | -0.012 | 1.000 |  |  |  |  |  |  |  |  |  |  |
|  |  |  |  |  |  |  |  |  |  |  |  |  |
| **(3) ESG** | 0.007 | 0.278*** | 1.000 |  |  |  |  |  |  |  |  |  |
|  |  |  |  |  |  |  |  |  |  |  |  |  |
| **(4) MEDCO** | -0.039* | -0.309*** | -0.104*** | 1.000 |  |  |  |  |  |  |  |  |
|  |  |  |  |  |  |  |  |  |  |  |  |  |
| **(5) AUDTE** | 0.107*** | -0.018 | 0.005 | -0.090*** | 1.000 |  |  |  |  |  |  |  |
|  |  |  |  |  |  |  |  |  |  |  |  |  |
| **(6) BBS** | -0.003 | 0.32*** | 0.033 | -0.123*** | -0.048** | 1.000 |  |  |  |  |  |  |
|  |  |  |  |  |  |  |  |  |  |  |  |  |
| **(7) BGD** | 0.001 | -0.164*** | 0.027 | 0.033 | 0.000 | -0.031 | 1.000 |  |  |  |  |  |
|  |  |  |  |  |  |  |  |  |  |  |  |  |
| **(8) CSRAUD** | -0.006 | 0.298*** | 0.315*** | -0.228*** | 0.032 | 0.158*** | -0.015 | 1.000 |  |  |  |  |
|  |  |  |  |  |  |  |  |  |  |  |  |  |
| **(9) ROA** | 0.031 | -0.101*** | 0.007 | -0.001 | 0.047** | -0.012 | 0.042* | -0.003 | 1.000 |  |  |  |
|  |  |  |  |  |  |  |  |  |  |  |  |  |
| **(10) FSIZE** | -0.030 | 0.665*** | 0.299*** | -0.205*** | -0.056** | 0.389*** | -0.178*** | 0.294*** | -0.003 | 1.000 |  |  |
|  |  |  |  |  |  |  |  |  |  |  |  |  |
| **(11) LEV** | -0.037* | 0.148*** | 0.009 | 0.020 | -0.044** | 0.053** | -0.117*** | 0.028 | -0.135*** | 0.287*** | 1.000 |  |
|  |  |  |  |  |  |  |  |  |  |  |  |  |
| **(12) CUR** | -0.016 | 0.044*** | 0.019 | 0.009 | -0.033 | 0.018 | 0.016 | 0.004 | -0.004 | 0.066*** | -0.045** | 1.000 |
| **Variance Inflation Factor (VIF)** | **1.01** | **1.99** |  | **1.15** | **1.03** | **1.20** | **1.05** | **1.15** | **1.05** | **2.15** | **1.14** | **1.01** |
| **1/VIF (Tolerance)** | **0.99** | **0.50** |  | **0.87** | **0.97** | **0.83** | **0.95** | **0.87** | **0.96** | **0.46** | **0.88** | **0.99** |

**ESG**: environmental, social and governance, **AUDFEE:** audit fees **MEDCO**: media coverage, **AUDTE**: audit tenure, **BBS**: Board background and skill, **BGD**: board gender diversity, **CSRAUD:** corporate social responsibility auditors, **ROA:** return on assets, **FSIZE:** firm size **CUR**: current ratio, **LEV**: leverage, *The estimated coefficient is statistically significant at ** p<.1*; **The estimated coefficient is statistically significant at *** p<.05*; ***The estimated coefficient is statistically significant at **** p<.01*
